# Priorities for developing countries in the global response to non-communicable diseases

**DOI:** 10.1186/1744-8603-8-14

**Published:** 2012-06-11

**Authors:** Dermot Maher, Nathan Ford, Nigel Unwin

**Affiliations:** 1 London School of Hygiene and Tropical Medicine, Keppel Street, London, England; 2 Médecins Sans Frontières, Geneva, Switzerland; 3 Centre for Infectious Disease Epidemiology and Research, University of Cape Town, Cape Town, South Africa; 4 Faculty of Medical Sciences, University of The West Indies Cave Hill Campus, Bridgetown, Barbados

**Keywords:** Non-communicable diseases, Prevention, Treatment

## Abstract

The growing global burden of non communicable diseases (NCDs) is now killing 36 million people each year and needs urgent and comprehensive action. This article provides an overview of key critical issues that need to be resolved to ensure that recent political commitments are translated into practical action. These include: (i) categorizing and prioritizing NCDs in order to inform donor funding commitments and priorities for intervention; (ii) finding the right balance between the relative importance of treatment and prevention to ensure that responses cover those at risk, and those who are already sick; (iii) defining the appropriate health systems response to address the needs of patients with diseases characterized by long duration and often slow progression; (iv) research needs, in particular translational research in the delivery of care; and (v) sustained funding to support the global NCD response.

## Background

The growing global non-communicable disease (NCD) crisis is now killing 36 million people each year and needs urgent and comprehensive action [1]. Rapidly increasing globalization is accompanied by urbanization, population growth and ageing, and trends towards unhealthy lifestyles, including unhealthy diets, physical inactivity, obesity, and immoderate alcohol and tobacco use. Chronic NCDs are defined by the World Health Organization (WHO) as cardiovascular diseases (CVD), diabetes, chronic respiratory diseases, and cancer. This grouping of chronic NCDs reflects their association with common shared risk factors: harmful use of alcohol, tobacco use, physical inactivity and unhealthy diets [2]. Non-communicable diseases in low-income and middle-income countries currently account for 80% of the worldwide NCD mortality caused by CVD, cancers, diabetes and chronic lung diseases [3].

Attention to NCDs is increasing for several reasons. First country-level data show that these conditions are contributing to epidemiological transition in a wide range of low- and middle-income countries [3]. Second, these data support estimated disease projections of a growing burden of morbidity and mortality associated with chronic NCDs. Third, NCDs have a huge negative economic impact [4] and represent a significant impediment to human development [5]. Fourth, recent progress in mobilising funds and improving the response to infectious diseases (especially HIV/AIDS, tuberculosis and malaria) has stimulated a broader global health outlook. These factors culminated in a United Nations (UN) High-Level Meeting on NCDs in New York in September 2011 [6].

A worldwide goal for the prevention and control of NCDs has been proposed to complement existing MDG targets for communicable disease control, with the accompanying target of an additional 2% per year reduction in death rates attributable to the main chronic diseases (heart disease, stroke, cancer, diabetes, and chronic respiratory diseases) [7]. Achieving this goal will require comprehensive action covering the range of diseases and risk factors through a two-pronged approach: implementation of the multisectoral policies aimed at decreasing population-level risks for NCDs, and effective and affordable delivery of health sector interventions for patients with NCDs.

Feasible and cost-effective health sector interventions exist for the priority chronic NCDs. For example, glycaemic control, hypertension control, and foot care in people with a high risk of ulcers have been ranked as priority low-cost interventions for diabetes control [8]. For the management of individuals with chronic vascular disease cost-effective interventions include treatments with low cost generic drugs, lifestyle and behavioural changes, and rehabilitative measures [9]. The potential impact of scaling up evidence-based health sector interventions for the prevention of NCDs in low-income and middle-income countries is considerable. As an example, scaling up a multidrug regimen (the ‘polypill’, i.e. a statin, aspirin, and two antihypertensive drugs) for the prevention of CVD in high-risk individuals could avert 18 million deaths over a 10-year period, at an average yearly cost per head of $1.08 [10]. The identification of simple, low-cost interventions is all the more important for developing countries, which generally have under-resourced health-systems, particularly in rural areas.

This paper will address a number of key policy issues that will need to be addressed to enable an effective response to NCDs in low- and middle-income countries.

## Key policy issues

### Categorizing and prioritizing NCDs

Given the enormously broad range of diseases which are non-communicable it is necessary to group certain diseases for certain purposes. Whichever grouping of chronic NCDs is used for a particular purpose, it is important to specify the criteria for the inclusion of the particular NCDs in that grouping.

The definition of the priority NCDs – CVD, diabetes, chronic respiratory diseases, and cancer - by WHO is based on their shared risk factors and therefore a common approach to their prevention. Within the individual disease categories there are also groupings of diseases linked by shared risk factors and therefore amenable to similar approaches for their control. For example, within the group of CVDs, the primary and secondary prevention of ischaemic heart disease and ischaemic cerebrovascular disease (e.g. ischaemic stroke and transient ischaemic attacks) rely on similar measures, including a healthy diet, use of aspirin and statins, smoking cessation, and hypertension control. The focus of WHO efforts is to try to ensure that the priority NCDs are urgently accorded greater attention in the health and development policies of poor nations and on global aid agendas. Recent efforts include the launch of the new global initiative in July 2009 [11] and the WHO contribution to the UN high-level meeting on NCDs in September 2011 [12].

As a group of chronic NCDs, CVD, diabetes, and chronic respiratory diseases share the following specific characteristics: 1) they can be detected using simple tests available (or potentially readily available) in primary care settings in low-income countries: hypertension (sphygmomanometer), chronic airflow obstruction (peak expiratory flow meter), diabetes (urine or blood glucose) and obesity (weight and height); 2) they can be readily managed in typical primary care settings in middle- and low-income countries; 3) the benefits of prevention and care extend to related conditions of public health importance, e.g. chronic kidney disease (often caused by hypertension or diabetes). These shared characteristics are the basis for inclusion of these diseases in a proposed framework for NCD primary care in developing countries [13]. The approach to each NCD has its own specific set of challenges, and cancer in particular represents a special case. Since capacity for managing people with cancer in many developing countries, especially in Africa, is very limited and invariably centralised [14] the health-care system response to people with cancer involves in some ways a greater challenge than the response to people with CVD, diabetes, and chronic respiratory diseases.

The implications of a particular disease being included on a list for an international agenda are crucial. For example, the grouping of HIV, tuberculosis and malaria as “priority diseases of poverty” had immense implications for funding, as these specific diseases became the beneficiaries of increased international funding through the establishment of the Global Fund to Fight AIDS, Tuberculosis and Malaria.

Depending on the purpose of grouping a set of chronic NCDs in a particular category, there may be leeway for a flexible approach to grouping. For example, the initial grouping of NCDs in the framework for a structured approach to the prevention and management of chronic NCDs in primary care can potentially be extended to include other chronic NCDs such as chronic kidney disease, chronic liver disease and chronic organic brain syndromes (e.g. the dementias). One area of recent contention has been the relegation of psychiatric conditions to a second ranking in the list. For example, at the First Global Ministerial Conference on NCDs and Healthy Lifestyles held in Moscow from 28–29 April 2011, mental disorders were included in the declaration under the “other NCD” category: “In addition, other NCDs such as mental disorders also significantly contribute to the global disease burden” [15]. This is in contrast to disease projections which put mental health disorders at the top of the global disease burden league table by 2030.

### Relative importance of prevention and care

There is debate about the relative importance of prevention and care regarding the common priority chronic NCDs as defined by WHO. In the run-up to the UN high-level meeting the focus of discussions in leading medical journals on priority actions within the health sector mainly focused on the multisectoral policies for prevention of NCDs – policies which lie beyond the health sector and mainly require government action, such as tobacco and alcohol control, ensuring environmental safety, and promotion of agricultural and food industry reforms [16,17]. Multisectoral actions for prevention are necessary to reduce the future disease burden, but must be accompanied by health sector actions for care, considering that it is the current disease burden that is essentially being used to justify the investments mainly aimed at prevention. One of the lessons learned from previous pioneering UN summits on HIV is the importance of agreeing on priority actions for both prevention and care, which may be mutually reinforcing [18]. For example, meeting the care needs of people with NCDs may enhance the impact of prevention policies, since people are more likely to test for a disease for which there is a good prospect of effective treatment. Conversely, in populations where up to 30% of adults may have a chronic NCD, prevention policies that support healthier diets, more physical activity, less smoking and less harmful use of alcohol create an environment which facilitates the adoption of these lifestyle measures which are a key part of the management of conditions such as diabetes, hyptertension, CVD and chronic respiratory disease.

In the present era of global economic crisis and fiscal restraint, governments are exploring ways of decreasing expenditures on social sectors, including health [19]. Arguments for prevention based on economic benefits are likely to find a more sympathetic ear among governments and international funders than arguments for increased spending on care. Chronic NCDs by definition are not curable and so patients require lifelong care. It may be difficult to convince funders and some elements of the healthcare professions of the benefits of investing in detection and treatment of chronic NCDs when some are asymptomatic and the benefit for individuals of detection and treatment is expressed as a likelihood rather than certainty, e.g. hypertension. However, there are enough data showing that the impact of NCDs such as hypertension, CVD and diabetes is similar in different parts of the world, and the benefits of investment in care of people with NCDs are just as likely to apply in developing countries as in developed countries. Any population differences in disease manifestations and responses to treatment need to be taken into consideration in planning and implementing health care delivery rather than used as a potential argument against NCD care delivery in certain populations. This is the case in developed countries, where health care delivery is adapted to meet the specific needs of people whose origin in developing countries is associated with population-specific differences in NCD progression and presentation, e.g. increased risk of type 2 diabetes and subsequent CVD in people of south Asian origin.

There may be some unwillingness among the international health community to take on the enormous task of providing quality lifelong care for people with NCDs at a time when a sense of “combat fatigue” is becoming apparent in relation to the effort to provide lifelong treatment for people with HIV infection. This effort is slackening off even before the task is half completed [20]. It may be the case that the health sector experts’ emphasis on priorities for action outside the health sector represents an unspoken unwillingness to embrace the challenge of large scale action in the health sector for people with NCDs. Just as pioneering projects by Médecins sans Frontières (MSF) and others changed the mindset that “HIV treatment in developing countries is too difficult” [21], similar pioneering projects may well be needed to show that the mindset that “NCD treatment on a massive scale in developing countries is too difficult” is untenable.

As the global community gears up towards actions and investments aimed at limiting the number of future potential NCD patients, the voice of health advocates representing the needs of people with NCDs for quality care today and tomorrow needs to find its place. A balance must be found between the upstream, multisectoral policies for NCD prevention aimed at the benefit of future generations, and the downstream, health sector interventions for ensuring that people with NCDs obtain quality care.

### The health system response to NCDs in developing countries

The inverse care law applies to the global problem of NCDs: with 80% of global NCD deaths occurring in low and middle-income countries, those most in need of care have least access. The need to improve the health system response in developing countries is greatest in sub-Saharan Africa, which faces the greatest predicted increase in NCD deaths [11] and has the least resources for an effective response to the double burden of communicable diseases and NCDs. Health systems in developing countries have often been orientated towards tackling communicable disease, and the approach to NCDs is often unstructured, lacks systematic follow-up and monitoring of chronic clinical care, and provides little information about morbidity or mortality, which is crucial for effective health planning [22]. There is therefore broad agreement that “Health systems need to be further strengthened to deliver an effective, realistic and affordable package of interventions and services for people with NCDs” [2]. The importance of overall strengthening of health systems is as relevant for an effective response to NCDs as to chronic communicable diseases such as HIV and tuberculosis [23]. Health systems also need to be reoriented to managing people with chronic conditions. The structure of these health systems generally reflects a model of health care response to acute episodes of illness. The increasing burden of chronic disease in low-income countries poses a challenge in reorienting health systems so that they can address the needs of patients with diseases characterised by long duration and often slow progression [24]. The progress made in developing successful ways of managing chronic infectious diseases, including community-based support, such as tuberculosis and HIV infection is proof of principle that a reoriented health-care system response to chronic diseases is possible.

### Role of primary care

The scale of the burden of NCDs and the cost implications point towards the importance of a response to NCDs centred on primary care rather than hospitals at secondary or tertiary level [24]. The importance of a strong health system led by primary care is receiving renewed attention [25]. Primary-care providers include those in the government services (Ministry of Health, social-security, prisons, military) and nongovernment services (nongovernmental organizations and private practitioners). In practice, secondary and tertiary care institutions often also provide primary care in addition to playing a referral role. Primary-care delivery in sub-Saharan Africa has shortcomings: it may be impoverishing, fragmented, unsafe or misdirected [26]. However, a key strength of primary care is that it is the main entry point into health services for most people. It has played a successful role in the delivery of prevention and care interventions for communicable diseases such as tuberculosis, HIV and malaria. Building on this success, primary care could potentially play a key role in the delivery of prevention and care interventions [27]. Investing in improved primary care has the potential to overcome some of the problems identified with the current health system approach to NCDs [28]. The practical policy proposals to improve the primary care response to the problem of NCDs in developing countries undergoing health transition include the following: (i) improving data on communicable and non-communicable diseases; (ii) implementing a structured approach to the improved delivery of primary care; (iii) putting the spotlight on quality of clinical care; (iv) aligning the response to health transition with health system strengthening; and (v) capitalizing on a favourable global policy environment [28].

### The need for a public health approach to care of people with NCDs

At present the health system approach to NCDs in developing countries is generally on an individual basis, i.e. a patient consults a care provider who delivers health interventions. This individual approach to care is in contrast to a public health approach in which a programmatic structure enables systematic follow-up, monitoring of the quality of care, and routine collection and reporting of information about patient outcomes, morbidity and mortality (information which is crucial for effective health planning). A focus on quality care is key in bridging clinical care and public health [29].

It is useful to review briefly the development of the public health approach to provision of ART for people with HIV infection to provide a concrete example of a public health approach to care in action. Based on extensive evidence that ART can substantially extend the life of people with HIV infection, guidelines for industrialised countries covered individual patient management delivered by specialist doctors prescribing from a wide range of ARVs supported by routine high-technology laboratory monitoring. Such an approach is not feasible in resource-limited settings where doctors are scarce, laboratory infrastructure is inadequate, and the procurement and supply-chain management is fragile. The difficulty in translating the guidelines from developed to developing nations caused concerns over whether ART scale-up in poor countries was feasible, let alone affordable or cost-effective.

Drawing on the experience of the internationally recommended tuberculosis control strategy [30], a public-health approach to providing ART was developed [31]. This approach took into account country requirements, the realities of weak health systems, and the experiences of pioneering ART programmes such as those of Médecins sans Frontières. Standardisation of diagnosis and treatment and simplification of regimens support efficient implementation [32]. A structured public health approach promotes implementation of evidence-based programmes and equity (setting standards for treatment that should be accessible by all in need). The move from an individual-based approach to a population-based one was recognised as the only way to make ART rapidly accessible to the millions in need and was a key conceptual shift [33]. The public health approach to the delivery of individual health interventions has enabled substantial, if still incomplete, progress in rolling out interventions for the diagnosis and treatment of tuberculosis [34] and HIV infection [35].

Although a public health approach to the delivery of individual health interventions is crucial to providing wide access to quality care, these lessons have not so far been applied for the benefit of people with NCDs. For example, the WHO global NCD status report describes the NCD burden and inadequacies of the current health system response but gives weak guidance about how interventions can be delivered [2]. The report dedicates a chapter to “Individual Health Interventions” without addressing need for a public health approach to the delivery of “individual health interventions” [2].

The strategy for addressing the needs of people with NCDs in a public health approach has three key elements: identifying and addressing modifiable risk factors; screening for common NCDs; and diagnosing, treating, following up and, when necessary, referring patients with common NCDs using standard protocols [13]. The package of interventions for quality care comprises political commitment, case-finding among people attending primary care services, standardised diagnostic and treatment protocols, regular drug supply, and systematic monitoring and evaluation. Given the enduring crisis in human resources, task shifting, community engagement, and the enrolment of expert patients are all likely to be critical - a lesson learned from the HIV experience [36].

There is an urgent need to evaluate such an approach in the field and if found successful to extend it on a wide scale, analagous to massive scale-up of the international strategy for tuberculosis control and of the public health approach to ART access [37]. A focus on providing quality care can provide a link between clinical and public health approaches to NCDs as shown for HIV infection [29]. Sub-optimal care for many people with chronic disease is a problem worldwide, e.g. hypertension and diabetes are poorly controlled in a high proportion of patients even in developed countries. The lessons learned from using a structured public health approach to improve on a large scale the quality of care for people with chronic diseases in developing countries may also be relevant to countries in the developed world, especially those where the poor and disadvantaged have limited access to ongoing healthcare provision (e.g. USA).

One key lesson from both HIV care in developing countries and NCD care in developed countries is the need for policies and approaches that make care more patient centered, including shared decision-making, and promote patient activation and empowerment. These important lessons should be integral to NCD care in resource-limited settings where access to health centres and providers is limited, and the potential contribution of patients as co-providers of care is all the more important.

### Interaction between communicable diseases and NCDs

An increasing disease burden arises from interactions between communicable diseases and NCDs [38], e.g. between tuberculosis and poor nutritional status and tobacco use, diabetes and infection (with diabetes predisposing to infections which often exacerbate hyperglycaemia). Common NCDs arising from the current high burden of chronic communicable diseases in Africa include cervical cancer linked to human papilloma virus infection and hepatoma linked to hepatitis B virus infection. The burden of chronic NCDs is likely to be further uncovered as scaled-up programmes of antiretroviral treatment of HIV-infected people reduce mortality but increase morbidity related to chronic HIV infection and treatment. Increasing numbers of people in Africa are therefore at risk of possible metabolic side effects resulting from life-long antiretroviral treatment [35], e.g. diabetes, lipodystrophy and dyslipidaemia. These overlaps between communicable diseases and NCDs present opportunities for synergistic care within the context of strengthened health systems [38].

At least 2 million cancer cases per year (18% of the global cancer burden) are attributable to a few specific chronic infections, and this fraction is substantially larger in low-income countries [2]. The principal infectious agents are human papillomavirus (cervical cancer), hepatitis B virus and hepatitis C virus (liver cancer) and *Helicobacter pylori* (gastric cancer). These infections are largely preventable through vaccinations and measures to avoid transmission, or are treatable. For example, transmission of hepatitis C virus has been largely stopped among high-income populations, but remains a problem in many low-resource countries [2].

### Ensuring access to affordable diagnostics and drugs

An essential lesson from the scale-up of HIV treatment is that a concerted international effort is required to bring down the cost of essential diagnostics and drugs, since many essential drugs for NCDs are unaffordable and consequently unavailable in many resource-limited settings [39]. In response to the often limited access to anti-asthma drugs provided at a relatively high cost [40] the Asthma Drug Facility was established to promote the supply of drugs for asthma and may be further extended to drugs for other NCDs [41]. The model drug supply system for the Asthma Drug Facility is the Global Drug Facility for anti-TB drugs [42]. In the case of antiretroviral drugs for HIV/AIDS, the most important driver of affordable treatment was the introduction of generic competition that drove down the global cost of treatment by over 90% within just a few years [43]. Thus different approaches are available to policy makers to drive down drug prices and increase access, and these should be considered early in the NCD response.

Ensuring reliable drug supply is a critical area and again there are lessons from HIV. Considerable efforts have been made to reinforce drug forecasting, support supply chain management, and rational use of antiretrovirals, supported by organizations such as The Clinton Health Access Initiative and UNITAID. These lessons too should be applied in the scale up of NCD treatment access.

Finally, access to essential diagnostics should not be neglected. Viral load has been recognized as an important tool in the clinical and programme management of HIV yet a decade into the scale up response, viral load remains too complex and costly for the majority of high HIV-burden countries [44].

### Priority research needs

Since there are enough data to show that the impact of NCDs such as hypertension and diabetes is essentially similar in different populations around the world, the priority research need is for operational research in the delivery of care [45]. So what should be delivered is, to a considerable degree, well known, but how to deliver it effectively in low-resource settings is much less well known. Basic epidemiology provides needs assessment in establishing the population burden of NCDs, and then highly pragmatic translational intervention studies enable validation and adaptation of the package of available interventions which are known to be cost-effective and the public health framework for delivering it. Where there is incomplete information on NCD epidemiology, presentation, progression and response to treatment, research is needed to fill the gaps [37]. For example, the sorts of large prospective studies on epidemiology, prognosis and treatment outcome of hypertension that have been conducted in developed countries need to be conducted in developing countries. Studies of prognosis can be conducted in the context of learning about how to deliver care.

A crucial issue is that limited research is currently being conducted on chronic NCDs in developing countries, especially in sub-Saharan Africa, and resources for such research are severely limited. Overcoming this problem requires both increased investment and more efficient use of resources. The main international research funders have shown some signs of increased interest in funding research on NCDs, but with a focus on the large emerging economies such as China and India [46]. One way of using research resources more efficiently is to identify existing research infrastructures established for other purposes and use them for research on NCDs [47]. This avoids incurring the costs of establishing an entirely new research infrastructure specifically for NCDs in countries with severely constrained resources. An example of the success of this approach is the use of a research infrastructure in rural Uganda initially established for survey of HIV infection as a platform for survey of hypertension and other chronic NCDs provides [48,49].

Arguments for investment in disease prevention and management can be based on promoting health as a human right and as a contributor to poverty reduction and economic stability. The economic case was made successfully in the past for tuberculosis [50] and more recently for HIV [51], and now needs to be developed for investment in a two-pronged approach to the global problem of NCDs: the upstream multisectoral actions for prevention, and the complementary downstream health sector actions for care. Detailed analyses have been done on funding for other priority global health problems such as HIV, tuberculosis and malaria. Research is needed to track the financing of delivery of health care also for people with NCDs, involving external donor funding and domestic funding by national governments. Crucial questions include the sources, extent and distribution of international funding for NCDs.

### Global funding

Despite increasing recognition in some quarters of the double burden in developing countries of chronic communicable diseases and chronic NCDs [52], health funders have not yet mobilized the substantial investment required to respond to the challenge of hypertension and other chronic NCDs globally, including in Africa [53]. Recent progress in mobilizing funds and improving the response to infectious diseases (especially HIV/AIDS, tuberculosis and malaria) now enables a shift to a broader global health outlook, for example, there is growing discussion about how the Global Fund To Fight Aids, Tuberculosis And Malaria can drive broad improvements in general health systems, which should also include a focus on NCDs [54]. New alliances, such as the Global Alliance for Chronic Diseases, may provide an opportunity for concerted action to put the evidence base for treatment and control of NCDs in developing countries on a firm footing. The challenge is to ensure that funding streams for chronic diseases are sustained over time. The sustainability of the current, largely externally funded approach to provide universal access to ART is currently under threat due to faltering donor commitments in the enduring global economic crisis. This should provide sober reflection to the enthusiasm around current political commitments to do more for NCDs: if these commitments are serious, funding must follow, and it must be sustained.

## Conclusion

The recent political recognition of the importance of the global burden of non-communicable diseases is welcome, but it remains to be seen how political commitments will translate into practical action, particularly in resource-limited settings where the challenges of providing adequate care and treatment are significant. Much can be learnt from the last decades’ struggle to improve access to HIV treatment and care [55]. In particular, the need to balance treatment and prevention, rather than pit one against the other, needs to be addressed from the outset.

## Competing interests

The authors declare that they have no competing interests.

## Authors’ contributions

DM wrote the first draft of the manuscript. All authors contributed to revising subsequent drafts and approved the final version.

## References

[B1] World Health Organization Global infobasehttps://apps.who.int/infobase/mortality.aspx2

[B2] World Health Organization Global status report on non-communicable diseases 2010 http://whqlibdoc.who.int/publications/2011/9789240686458_eng.pdf

[B3] Geneau R Stuckler D Stachenco S Raising the priority of preventing chronic diseases: a political process Lancet 2010 376 1689 1698 21074260 10.1016/S0140-6736(10)61414-6

[B4] Suhrcke M Nugent RA Stuckler D Rocco L Chronic disease: an economic perspective 2006 Oxford Health Alliance, London

[B5] Adeyi O Smith O Robles S Public policy and the challenge of chronic non-communicable diseases 2002 World Bank, Washington, DC

[B6] General Assembly of the United Nations High-level Meeting on Non-communicable Diseaseshttp://www.un.org/en/ga/president/65/issues/ncdiseases.shtml

[B7] Strong K Mathers C Leeder S Beaglehole R Preventing chronic diseases: how many lives can we save? Lancet 2005 366 1578 1582 16257345 10.1016/S0140-6736(05)67341-2

[B8] Narayan KMV Zhang P Kanaya AM Jamison DT, Breman JG, Measham AR Diabetes: the pandemic and potential solutions Disease control priorities in developing countries 2006 2 Washington, DC, The World Bank 21250309

[B9] Gaziano TA Reddy KS Paccaud F Horton S Chaturvedi V Jamison DT, Breman JG, Measham AR Cardiovascular disease Disease control priorities in developing countries 2006 2 Washington, DC, The World Bank 21250309

[B10] Lim SS Gaziano TA Gakidou E Prevention of cardiovascular disease in high-risk individuals in low-income and middle-income countries: health effects and costs Lancet 2007 370 2054 2062 18063025 10.1016/S0140-6736(07)61699-7

[B11] World Health Organization 2008–2013 Action Plan for the Global Strategy for the Prevention and Control of Noncommunicable Diseases 2008 WHO, Geneva

[B12] World Health Organization Sixty-fourth World Health Assembly. Document A64/21 2011 WHO, Geneva

[B13] Maher D Harries A Zachariah R Enarson D A global framework for action to improve the primary care response to chronic non-communicable diseases: a solution to a neglected problem BMC Publ Health 2009 9 355 10.1186/1471-2458-9-355 PMC2758871 19772598

[B14] Farmer P Frenk J Knaul FM Expansion of cancer care and control in countries of low and middle income: a call to action Lancet 2010 376 1186 1193 20709386 10.1016/S0140-6736(10)61152-X

[B15] First Global Ministerial Conference on NCDs and Healthy Lifestyles 2011 http://www.who.int/nmh/events/moscow_ncds_2011/en/

[B16] Piot P Ebrahim S Prevention and control of chronic diseases Br Med J 2010 342 293 294 10.1136/bmj.c4865 21078750

[B17] Beaglehole R Bonita R Horton R Priority actions for the non-communicable disease crisis Lancet 2011 377 1438 1447 21474174 10.1016/S0140-6736(11)60393-0

[B18] General Assembly of the United Nations High-level Meeting on AIDS 2006 http://www.un.org/ga/aidsmeeting2006

[B19] Maher D Implications of the global financial crisis for the response to diseases of poverty within overall health sector development: the case of tuberculosis Trop Med Int Health 2010 15 1 11 17 19891762 10.1111/j.1365-3156.2009.02412.x

[B20] Maher D von Schoen-Angerer T Cohn J Crunch time for funding of universal access to antiretroviral treatment for people with HIV infection Int J Clin Pract 2011 65 824 827 21762306 10.1111/j.1742-1241.2011.02697.x

[B21] Bemelmans M van de Akker T Ford N Philips M Zachariah R Harries A Schouten E Hermann K Mwagomba B Massaquoi M Providing universal access to antiretroviral therapy in Thyolo, Malawi through task shifting and decentralization of HIV/AIDS care Trop Med Int Health 2010 12 1413 1420 20958897 10.1111/j.1365-3156.2010.02649.x

[B22] Wilkinson D Wilkinson NF Parry E, Godfrey R, Mabey D, Gill G Organisation of non-communicable disease care Principles of medicine in Africa 2004 3 Cambridge University Press, Cambridge

[B23] Maher D Re-thinking global health sector efforts for HIV and tuberculosis epidemic control: promoting integration of programme activities within a strengthened health system BMC Publ Health 2010 10 394 10.1186/1471-2458-10-394 PMC3091552 20602774

[B24] Allotey P Reidpath DD Yasin S Chan CK Aikins AD Rethinking health-care systems: a focus on chronicity Lancet 2011 377 450 451 21074257 10.1016/S0140-6736(10)61856-9

[B25] Rawaf S De Maeseneer J Starfield B From Alma-Ata to Almaty: a new start for primary health care Lancet 2008 372 1365 1367 10.1016/S0140-6736(08)61524-X 18922572

[B26] World Health Organization The world health report 2008: primary health care, now more than ever 2008 WHO, Geneva

[B27] Jha P Control of non-communicable diseases in sub-Saharan Africa Disease Control Priorities Project.http://www.dcp2.org/file/203/djcc_jha.pdf

[B28] Maher D Smeeth L Sekajugo J Health transition in Africa: practical policy proposals for primary care Bull World Health Organ 2010 88 943 948 21124720 10.2471/BLT.10.077891 PMC2995191

[B29] Maher D Harries AD Quality care: a link between clinical and public health approaches to HIV infection in developing countries Trop Med Int Health 2010 15 4 391 395 20180935 10.1111/j.1365-3156.2010.02472.x

[B30] Maher D Kaufmann S, Rubin E, Britton W, Helden P Tuberculosis control: good clinical care and good public health Tuberculosis handbook 2008 Mannheim, Germany, Wiley-VCH

[B31] World Health Organization Antiretroviral therapy for HIV infection in adults and adolescents 2010 WHO, Geneva http://whqlibdoc.who.int/publications/2010/9789241599764_eng.pdf 23741771

[B32] Maher D Standardised interventions in international health: Procrustes, where are you now? Trop Med Int Health 2009 14 11 1 3 19708901 10.1111/j.1365-3156.2009.02363.x

[B33] Gilks CF Crowley S Ekpini R Gove S Perriens J Souteyrand Y Sutherland D Vitoria M Guerma T de Cock K The WHO public-health approach to antiretroviral treatment against HIV in resource-limited settings Lancet 2006 368 505 510 16890837 10.1016/S0140-6736(06)69158-7

[B34] World Health Organization Global tuberculosis control: WHO Report 2010 2010 WHO, Geneva http://whqlibdoc.who.int/publications/2010/9789241564069_eng.pdf

[B35] World Health Organization Towards universal access: scaling up priority HIV/AIDS interventions in the health sector: progress report 2010 2010 http://www.who.int/hiv/pub/2010progressreport/en/

[B36] Joint United Nations Programme on HIV/AIDS Chronic care for HIV and non-communicable diseases. How to leverage the HIV experience 2011 UNAIDS, Geneva http://www.unaids.org/en/media/unaids/contentassets/documents/unaidspublication/2011/20110526_JC2145_Chronic_care_of_HIV.pdf

[B37] Maher D Sekajugo J Harries AD Grosskurth H Research needs for an improved primary care response to chronic non-communicable diseases in Africa Tropical Medicine and International Health 2010 15 2 176 181 20002618 10.1111/j.1365-3156.2009.02438.x

[B38] Young F Critchley JA Johnstone LK Unwin NC A review of co-morbidity between infectious and chronic disease in Sub Saharan Africa: TB and diabetes mellitus, HIV and metabolic syndrome, and the impact of globalization Global Health 2009 5 9 10.1186/1744-8603-5-9 PMID:19751503 19751503 10.1186/1744-8603-5-9 PMC2753337

[B39] Mendis S Fukino K Cameron A The availability and affordability of selected essential medicines for chronic diseases in six low- and middle-income countries Bull World Health Organ 2007 85 279 288 17546309 10.2471/BLT.06.033647 PMC2636320

[B40] Ait-Khaled N Auregan G Bencharif N Mady Carmara L Dagli E Djankine K Keita B Ky C Mahi S Ngoran K Pham DL Sow O Yousser M Zidouni N Enarson DA Affordability of inhaled corticosteroids as a potential barrier to treatment of asthma in some developing countries Int J Tuberc Lung Dis 2000 4 268 271 10751075

[B41] Asthma Drugs Facility http://www.globaladf.org/

[B42] Global Drug Facility for anti-tuberculosis drugshttp://www.stoptb.org/gdf/

[B43] Ford N Calmy A Mills E The first decade of antiretroviral therapy in Africa Glob Heal 2011 7 33 10.1186/1744-8603-7-33 PMC3192657 21958478

[B44] Ford N Roberts T Calmy A Viral load monitoring in resource-limited settings: a medical and public health priority AIDS 2012 [in press] 10.1097/QAD.0b013e3283543e2c 22874478

[B45] Zachariah R Ford N Maher D Bissel K van Den Bergh R van Den Boogaard W Is operational research delivering the goods? Assessing the journey from generating evidence to policy and practice change in low-income countries? Lancet Infect Dis 2012 12 415 421 22326018 10.1016/S1473-3099(11)70309-7

[B46] The global alliance for chronic diseases Lancet 2009 373 2084 10.1016/S0140-6736(09)61129-6 19541021

[B47] Maher D Sekajugo J Research on health transition in Africa: time for action Health Research, Policy and Systems 2011 9 5 21272381 10.1186/1478-4505-9-5 PMC3038153

[B48] Maher D Waswa L Baisley K Karabarinde A Unwin N Grosskurth H Distribution of hyperglycaemia and related cardiovascular disease risk factors in low-income countries: a cross-sectional population-based survey in rural Uganda Int J Epidemiol 2010 1 12 10.1093/ije/dyq156 20926371 PMC3043279

[B49] Maher D Waswa L Baisley K Karabarinde A Unwin N Epidemiology of hypertension in low-income countries: a cross-sectional population-based survey in rural Uganda J Hypertens 2011 29 1061 1068 21505357 10.1097/HJH.0b013e3283466e90

[B50] World Bank World development report 1993: investing in health 1993 Oxford University Press, New York

[B51] Schwartländer B Stover J Hallett T Atun R Avila C Gouws E Towards an improved investment approach for an effective response to HIV/AIDS Lancet 2011 early online publication 10.1016/S0140-6736(11)60702-2 21641026

[B52] World Health Organization The world health report 2004: changing history 2004 WHO, Geneva

[B53] Nugent RA Yach D Feigl AB Noncommunicable diseases and the Paris Declaration Lancet 2009 374 784 785 19733778 10.1016/S0140-6736(09)61589-0

[B54] Ooms G Van Damme W Baker BK Zeitz P Schrecker T The ‘diagonal’ approach to Global Fund financing: a cure for the broader malaise of health systems? Global Health 2008 4 6 18364048 10.1186/1744-8603-4-6 PMC2335098

[B55] Mills EJ Ford N Political lessons from the global HIV/AIDS response to inform a rapid non-communicable disease response AIDS 2012 26 1171 1173 22112604 10.1097/QAD.0b013e32834f3319

